# Glycyrrhizin preparations in liver diseases: a narrative review of mechanisms and therapeutic potential

**DOI:** 10.3389/fphar.2026.1795174

**Published:** 2026-04-30

**Authors:** Tianyu Ma, Hongxiao Hao, Shuojie Wang, Dianya Qiu, Weihua Cao, Wen Deng, Shiyu Wang, Xinxin Li, Ziyu Zhang, Xin Wei, Linmei Yao, Zixuan Gao, Wei Yi, Ruyu Liu, Minghui Li

**Affiliations:** 1 Department of Hepatology Division 2, Beijing Ditan Hospital, Capital Medical University, Beijing, China; 2 HBV Infection, Clinical Cure and Immunology Joint Laboratory for Clinical Medicine, Capital Medical University, Beijing, China; 3 Department of Gynecology and Obstetrics, Beijing Ditan Hospital, Capital Medical University, Beijing, China; 4 Department of Hepatology Division 2, Peking University Ditan Teaching Hospital, Beijing, China

**Keywords:** clinical applications, glycyrrhizin, glycyrrhizin preparations, liver disease, pharmacological mechanisms, safety

## Abstract

Glycyrrhizin preparations (GLPS), derived from Glycyrrhiza uralensis (licorice), are widely used hepatoprotective agents in clinical practice. Their primary active components, 18α- and 18β-glycyrrhetinic acid (GA), are represented by formulations including diammonium glycyrrhizinate (DG), compound glycyrrhizin (CG), and magnesium isoglycyrrhizinate (MgIG). This review provides a comprehensive overview of the pharmacokinetic properties of GLPS and their multi-target pharmacological mechanisms, comprising anti-inflammatory, membrane-stabilizing, antioxidant, anti-apoptotic, immunomodulatory, and anti-fibrotic effects that collectively underpin their hepatoprotective activity. We critically evaluate clinical evidence across major liver diseases, including viral hepatitis, drug-induced liver injury, alcoholic liver disease, non-alcoholic fatty liver disease, and autoimmune hepatitis, while highlighting heterogeneity in study designs, geographical concentration of evidence, and the predominance of positive findings that may reflect publication bias. Safety considerations, particularly the risk of pseudoaldosteronism and potential drug interactions, are also discussed. Finally, we outline future research directions, emphasizing the need for large-scale, multicenter randomized controlled trials with standardized outcomes to establish more robust evidence base and support broader international clinical adoption.

## 1 Introduction

**FIGURE 1:**
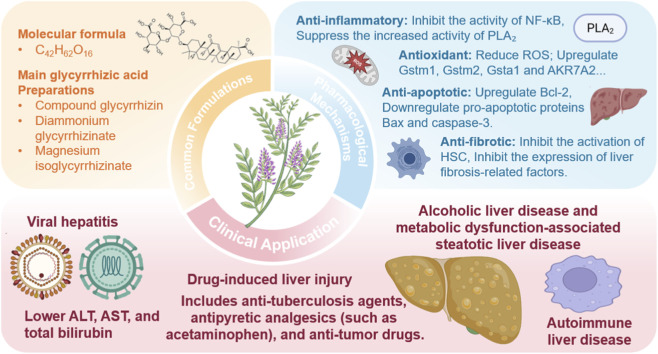
Pharmacological mechanisms and clinical applications of glycyrrhetinic acid preparations in liver disease therapy.

Liver disease represents a major global health challenge with diverse etiologies, including viral infections (hepatitis B and C), alcohol abuse, metabolic disorders (Metabolic Dysfunction-Associated Steatotic Liver Disease, MASLD), drug- and toxin-induced injuries, and autoimmune hepatitis. These factors commonly lead to hepatic inflammation, necrosis, and apoptosis, and persistent injury may progress to liver fibrosis, cirrhosis, and even hepatocellular carcinoma. Therefore, identifying agents capable of effectively suppressing liver inflammation and protecting hepatocyte function has long been a core objective in hepatology therapeutics. *Glycyrrhiza uralensis* Fisch., a traditional Chinese herb, has been used medicinally for thousands of years and is often referred to as “present in nine out of ten prescriptions” and honored as the “king of herbs” (Lee et al., 2015). With a sweet flavor and neutral nature, it is known for clearing heat, detoxifying, relieving cough and reducing phlegm, as well as harmonizing the effects of various medicinal ingredients (Lee et al., 2007). Modern pharmacological studies have revealed that glycyrrhizic acid (GL) and its metabolite glycyrrhetinic acid (GA), the core active components of licorice, are key substances responsible for its hepatoprotective effects (Chen et al., 2007; Kimura et al., 2001). For instance, GA mitigates inflammatory responses by modulating the production of inflammation-related molecules and enhancing the ability of hepatocytes to combat oxidative stress (Wang Q. et al., 2022). It has also been shown to ameliorate lipopolysaccharide-induced liver injury by regulating autophagy (Wu et al., 2021). Since the mid-20th century, various glycyrrhizin-based preparations (such as CG in Japan, and DG and MgIG in China) have been successfully developed and widely used in clinical practice, becoming foundational agents in the adjunctive treatment of liver diseases. This review aims to provide a comprehensive overview of the pharmaceutical properties and multi-dimensional pharmacological mechanisms of GLPS from a modern scientific perspective, summarize clinical evidence across different types of liver diseases, and objectively discuss their safety profiles, thereby providing a holistic overview to support rational clinical application and guide future research (Figure 1).

## 2 Chemical and pharmacokinetic characteristics of GLPS

### 2.1 Chemical structure and preparation types

**FIGURE 2:**
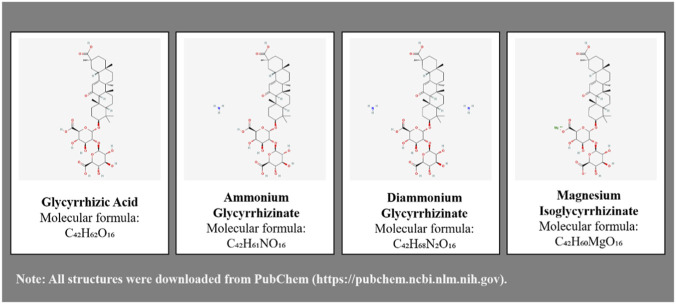
Chemical structures of major components in common glycyrrhizin preparations.

Glycyrrhizin, also known as GL, is a pentacyclic triterpenoid saponin and the primary active component of licorice (Xiao et al., 2024). It consists of one molecule of GA and two molecules of glucuronic acid (Zhang et al., 2023) (Figure 2). After ingestion, the aglycone moiety of glycyrrhizin is hydrolyzed by glucuronidase to form two stereoisomers, 18α-glycyrrhetinic acid (18α-GA) and 18β-glycyrrhetinic acid (18β-GA), which differ only in the configuration of the C18-H group (Chen et al., 2020). The α-isomer exhibits higher lipophilicity than the β-form, facilitating its binding to receptor proteins in the body (Chen et al., 2020). Naturally occurring licorice primarily contains the 18β-form, which demonstrates stronger biological activity. Several glycyrrhizin-based preparations are commonly used in clinical practice (Table 1).

**TABLE 1:** Commonly used glycyrrhizin preparations.

Common dosage form types	Composition	Solvent	References
Compound Glycyrrhizin	A mixture of 18α and 18β Composed of monoammonium glycyrrhizinate, L-glycine, and cysteine	5% GS or NS	Chen et al. (2020), Chen et al. (2024)
Diammonium Glycyrrhizinate	Ammonium salt form of glycyrrhizic acid Derived from the glycyrrhizin modified in the two carboxyl groups at the 6′and 6″position	150 mg + 10% GS	Chen et al. (2020), Sui et al. (2010)
Magnesium Glycyrrhizinate	Magnesium salt composed of 18α-glycyrrhetinic acid stereoisomers	100 mg + 10% GS	Chen et al. (2020), Fan et al. (2021)

CG typically consists of a mixture of 18α- and 18β-glycyrrhizin, often combined with glycine and cysteine to enhance efficacy and reduce side effects (Wen et al., 2021). It contains monoammonium glycyrrhizinate, glycine, and cysteine, and exerts broad pharmacological effects (Chen et al., 2024) (Figure 2). It is widely used clinically to treat drug-induced liver injury (DILI) by protecting hepatocyte membranes, exerting anti-inflammatory and immunomodulatory effects, and demonstrating corticosteroid-like actions that promote hepatocyte repair, enhance hepatic protein synthesis, prevent fibrosis, and strengthen anti-allergic and detoxification capacities. Its anti-inflammatory effect may be mediated through inhibition of phospholipase A2 activity (Wang, 2012).

DG, an ammonium salt form of glycyrrhizin, is derived from the glycyrrhizin modified in the two carboxyl groups at the 6′and six″position (Figure 2) (Sui et al., 2010). Compared to glycyrrhizin, it exhibits greater stability and higher biological activity (Chen et al., 2020; Zhu et al., 2012). DG possesses anti-inflammatory, antioxidant, anti-allergic, antiviral, and hepatoprotective properties and is clinically used to treat viral hepatitis and drug-induced liver injury (Zhao et al., 2017; Liu Y. et al., 2022). It also protects against alcohol-induced liver injury by modulating the DEAD-box helicase 5/Signal Transducer and Activator of Transcription 1 (DDX5/STAT1) signaling axis (Wang X. et al., 2024).

MgIG, a magnesium salt primarily composed of the 18α-glycyrrhizin stereoisomer, represents the fourth generation of GLPS (Fan et al., 2021). As a novel derivative extracted from traditional licorice, it demonstrates anti-inflammatory, antioxidant, antiviral, immunomodulatory, and hepatoprotective effects (Tang et al., 2021). In models of MASLD, MgIG protects the liver by regulating lipid metabolism (Jiang et al., 2020). In arsenic trioxide-induced acute liver injury models, it mitigates hepatotoxicity by suppressing oxidative stress, inflammation, and apoptosis (Liu M. et al., 2021) (Table 1).

### 2.2 Pharmacokinetics

**FIGURE 3:**
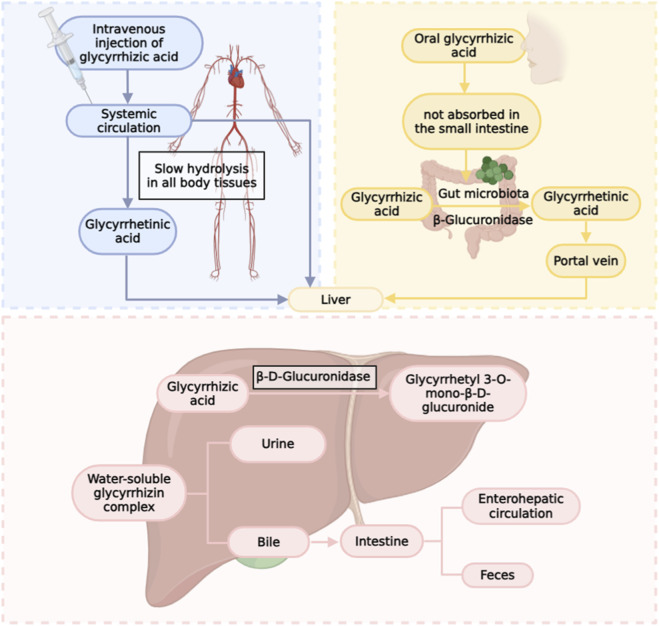
Metabolic pathways of glycyrrhizin in the body.

GLPS exhibit low oral bioavailability and are primarily administered intravenously for the treatment of acute liver injury. After oral administration, GL is hydrolyzed into GA in the intestine under the action of β-D-glucuronidase. GA is mainly transported to the liver via carriers and metabolized into glucuronic acid and sulfate conjugates. These conjugates are excreted into the duodenum via bile, where they are hydrolyzed back to GA by symbiotic bacteria, after which they can be reabsorbed (Ploeger et al., 2001). Following intravenous administration, GL is metabolized by β-D-glucuronidase in hepatic lysosomes to form 3-monoglucuronyl glycyrrhetinic acid (3MGA), which subsequently undergoes enterohepatic circulation (Chen et al., 2020). Ultimately, both GL and GA are primarily excreted as metabolites in bile and feces, with a small amount eliminated via the kidneys (Figure 3).

The pharmacokinetic (PK) profile of GL is highly dependent on the route of administration, with marked differences between oral and intravenous delivery due to its unique metabolic pathways and transporter-mediated disposition. In essence, GL acts as a prodrug that requires enzymatic conversion to its active metabolite, GA, which then undergoes extensive enterohepatic circulation.

Following oral administration, glycyrrhizin exhibits low oral bioavailability, primarily because it requires enzymatic hydrolysis by intestinal microbiota to generate its active metabolite, GA, which is then absorbed into the systemic circulation (Ploeger et al., 2001). Specific bacterial strains, including Eubacterium sp. (strain GHL), Ruminococcus sp. (PO1-3), and *Clostridium* innocuum (ES2406), possess specialized β-glucuronidases that hydrolyze GL to GA in the gastrointestinal tract, with optimal enzymatic activity occurring at pH values between 5.7 and 7.0 (Akao, 2000; Richard, 2021). After absorption, GA is transported to the liver primarily via carrier-mediated mechanisms and undergoes phase II metabolism to form glucuronide and sulfate conjugates (Chen et al., 2020; Ploeger et al., 2001). These conjugates are excreted into the duodenum via bile, where they are hydrolyzed back to GA by symbiotic bacteria, after which they can be reabsorbed, establishing an enterohepatic circulation that prolongs systemic exposure (Ploeger et al., 2001; Akao, 2000).

When administered intravenously, glycyrrhizin exhibits a biphasic plasma clearance profile, characterized by a rapid distribution phase followed by a slower elimination phase (Ploeger et al., 2001). GL is taken up from blood into hepatocytes primarily by human organic anion-transporting polypeptides OATP1B1 and OATP1B3 (Oatp1b2 in rats) (Ploeger et al., 2001). Within hepatocytes, GL is partially metabolized by lysosomal β-D-glucuronidase to form 3MGA (Chen et al., 2020). Both GL and 3MGA are subsequently transported into bile via canalicular efflux transporters, including multidrug resistance-associated protein 2 (MRP2), breast cancer resistance protein (BCRP), and bile salt export pump (BSEP) (Makino et al., 2008). Once excreted into the intestine, these compounds undergo further hydrolysis by gut microbiota to regenerate GA, which can then be reabsorbed, completing the enterohepatic circulation (Ploeger et al., 2001).

Irrespective of the administration route, GA predominates in systemic circulation and is rapidly and widely distributed throughout the body, with the highest concentration observed in liver tissue (Ploeger et al., 2001). Ultimately, both GL and GA and their metabolites are primarily eliminated via bile and feces, with only a small fraction excreted in urine (Figure 2) (Chen et al., 2020).

## 3 Core pharmacological mechanisms of glycyrrhizin in the liver

**FIGURE 4:**
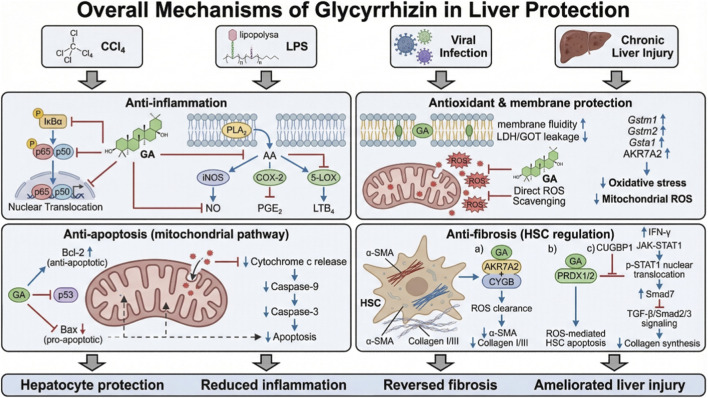
Schematic diagram of the hepatoprotective mechanisms of glycyrrhizin.

GA exerts hepatoprotective effects through multiple pathways and targets, forming a synergistic network that includes anti-inflammatory, antioxidant, liver-protecting, and immunomodulatory actions (Chen et al., 2020; Xie et al., 2015) (Table 2; Figure 4).

**TABLE 2:** Core pharmacological mechanisms of glycyrrhizic acid in the Liver.

Core function	Mechanisms	Key factors/Pathways	References
Anti-inflammation	1. Inhibition of NF-κB pathway	↓ IκBα phosphorylation, ↓ p65 nuclear translocation → ↓ iNOS, ↓ NO	Hsiang et al. (2015), Chen et al. (2014)
	2. Inhibition of arachidonic acid cascade	↓ PLA_2_ activity, ↓ COX-2/5-LOX expression → ↓ PGE_2_, ↓ PGI_2_, ↓ TXB_2_, ↓ LTB_4_	Xie et al. (2015)
Antioxidant and membrane protection	1. Membrane stabilization	Intercalation into phospholipid bilayer → ↑ membrane fluidity, ↓ LDH/GOT leakage	Selyutina et al. (2016a), Selyutina et al. (2016b), Nakamura et al. (1985), Selyutina and Polyakov (2019)
	2. Direct free radical scavenging	↓ ROS levels in mitochondria	Sil et al. (2016)
	3. Upregulation of antioxidant enzymes	↑ Gstm1, Gstm2, Gsta1 (glutathione S-transferase family); ↑ AKR7A2 (aldehyde clearance)	Wang et al. (2024b), Uys et al. (2014)
Anti-apoptosis	1. Regulation of mitochondrial apoptotic pathway	↑ Bcl-2, ↓ Bax → ↓ cytochrome c release → ↓ caspase-9/caspase-3 activation	Liang et al. (2015)
	2. p53-dependent pathway	↓ p53 activity → inhibition of mitochondrial apoptotic signaling	Liang et al. (2015)
Immunomodulation	1. Regulation of immune cell function	↑ T lymphocyte proliferation, ↑ macrophage iNOS/NO production	Cinatl et al. (2003)
	2. Antiviral effects	Suppression of viral gene expression/replication, modulation of host immune response	Pastorino et al. (2018), Sasaki et al. (2002)
Anti-fibrosis	1. nhibition of hepatic stellate cell (HSC) activation via oxidative stress modulation	Binding to AKR7A2 + CYGB → ↓ ROS in HSCs → ↓ α-SMA, ↓ collagen I/III synthesis	Wang et al. (2024b), Liang et al. (2015)
	2. Induction of HSC apoptosis via ROS	Binding to PRDX1/PRDX2 → ROS-mediated HSC apoptosis	Zhang et al. (2022)
	3. CUGBP1-IFN-γ-Smad7 axis	↓ CUGBP1 → ↑ IFN-γ → JAK-STAT1 → ↑ Smad7 → ↓ TGF-β/Smad2/3 signaling → ↓ collagen deposition	Guo et al. (2023); Wu et al. (2016); Miyazawa and Miyazono (2017); Liu et al. (2022b)
	4. Regulation of fibrosis-related factors	↓ CTGF, ↓ MMP2, ↓ MMP9 → reduced basement membrane disruption	Liang et al. (2015)

*Abbreviations*: AKR7A2, aldo-keto reductase family 7 member A2; Bcl-2, B-cell lymphoma 2; COX-2, cyclooxygenase-2; CTGF, connective tissue growth factor; CUGBP1, CUG-binding protein 1; CYGB, cytoglobin; GOT, glutamic-oxaloacetic transaminase; Gst, glutathione S-transferase; HSC, hepatic stellate cell; IFN-γ, interferon-gamma; IκBα, inhibitor of kappa B alpha; iNOS, inducible nitric oxide synthase; JAK, janus kinase; LDH, lactate dehydrogenase; LOX, lipoxygenase; LTB_4_, leukotriene B_4_; MMP, matrix metalloproteinase; NF-κB, nuclear factor kappa B; NO, nitric oxide; p53, tumor protein p53; PGE_2_, prostaglandin E_2_; PGI_2_, prostacyclin; PLA_2_, phospholipase A_2_; PRDX, peroxiredoxin; ROS, reactive oxygen species; Smad, mothers against decapentaplegic homolog; STAT1, signal transducer and activator of transcription 1; TGF-β, transforming growth factor-beta; TXB_2_, thromboxane B_2_; α-SMA, alpha-smooth muscle actin.

### 3.1 Modulation of inflammatory signaling pathways

#### 3.1.1 Inhibition of nuclear Factor-*κ*B (NF-*κ*B) pathway

Glycyrrhizin exerts anti-inflammatory effects and directly protects hepatocytes by regulating genes such as interleukin-4 (IL-4), fibroblast growth factor 10 (FGF10), inhibiting Inhibitor of kappa B (I*κ*B)α phosphorylation and p65 nuclear translocation to suppress NF-*κ*B activation in hepatocytes, thereby reducing the production of inducible nitric oxide synthase (iNOS) and nitric oxide (NO) (Hsiang et al., 2015; Chen et al., 2014) (Figure 4). Furthermore, in animal models, glycyrrhizin has been shown to inhibit the activation of hepatic stellate cells (HSCs) and induce their apoptosis by blocking NF-κB nuclear translocation, thereby preventing liver fibrosis (Qu et al., 2012).

#### 3.1.2 Interference with arachidonic acid cascade

In an lipopolysaccharide (LPS)-stimulated macrophage model, researchers found that MgIG inhibited the activation of PLA_2_ in LPS-stimulated macrophages (Xie et al., 2015). Phospholipase A_2_ (PLA_2_) is the key initiating enzyme of the arachidonic acid cascade, responsible for catalyzing the hydrolysis of membrane phospholipids to release arachidonic acid; therefore, MgIG restricts the arachidonic acid cascade at its source. Arachidonic acid is primarily metabolized via two pathways: the cyclooxygenase (COX) pathway and the lipoxygenase (LOX) pathway. This study demonstrated that MgIG significantly inhibited the protein expression of COX-2 and 5-LOX, key enzymes in both metabolic pathways of arachidonic acid induced by LPS (Xie et al., 2015) (Figure 4). Finally, experiments showed that MgIG suppressed the production of pro-inflammatory lipid mediators generated through the arachidonic acid metabolic pathway, including prostaglandin E_2_ (PGE_2_), prostacyclin (PGI_2_), thromboxane B_2_ (TXB_2_), and leukotriene B_4_ (LTB_4_) (Xie et al., 2015).

### 3.2 Direct hepatocyte protection: membrane stabilization and antioxidant effects

#### 3.2.1 Membrane stabilization

The amphiphilic structure of glycyrrhizin enables it to intercalate into the phospholipid bilayer of hepatocyte membranes, primarily in the outer layer. By carrying water molecules into the membrane, it increases membrane fluidity, facilitates the transmembrane transport of small molecules (e.g., ions and drugs), and under certain conditions, induces membrane thinning or even pore formation, thereby enhancing membrane permeability (Selyutina et al., 2016a; Selyutina et al., 2016b). In liver injury models induced by hepatotoxic substances such as carbon tetrachloride (CCl_4_), GA can suppress the abnormal increase in hepatocyte membrane permeability and reduce the leakage of lactate dehydrogenase (LDH) and glutamic-oxaloacetic transaminase, indicating its role in stabilizing the structure of hepatocyte membranes (Nakamura et al., 1985; Selyutina and Polyakov, 2019) (Figure 4).

#### 3.2.2 Free radical scavenging and antioxidant enzyme regulation

Glycyrrhizin functions as a free radical scavenger in preclinical models. In rodent studies, glycyrrhizin administration reduces reactive oxygen species (ROS) levels in liver mitochondria, indicating mitigation of mitochondrial oxidative stress (Sil et al., 2016). Proteomic analysis of liver tissue from CCl_4_ induced fibrosis models reveals that glycyrrhizin upregulates multiple members of the glutathione S-transferases family (Gstm1, Gstm2, Gsta1), which participate in glutathione metabolism and xenobiotic detoxification (Wang Q. et al., 2024; Uys et al., 2014). Additionally, glycyrrhizin directly binds to and upregulates aldo-keto reductase aldo-keto reductase family 7 member A2 (AKR7A2), an enzyme that clears aldehyde compounds produced by lipid peroxidation, thereby reducing ROS accumulation and oxidative stress-induced inflammation (Wang Q. et al., 2024).

### 3.3 Regulation of hepatocyte apoptosis

In liver injury, apoptosis is a major mode of hepatocyte death. The hepatoprotective effect of glycyrrhizin is partially achieved through the inhibition of hepatocyte apoptosis (Yan et al., 2016). In animal models, it upregulates the anti-apoptotic protein Bcl-2, downregulates the pro-apoptotic protein Bax, alters mitochondrial membrane permeability, and inhibits the activation of executioner caspases such as caspase-3, thereby blocking the apoptotic signaling pathway (Liang et al., 2015). GA can also suppress hepatocyte apoptosis via the p53-dependent mitochondrial pathway, further delaying the progression of liver fibrosis (Liang et al., 2015).

### 3.4 Immunomodulatory mechanisms

Glycyrrhizin exhibits multiple immunomodulatory mechanisms. It blocks the degradation of inhibitor of kappa B (IκB) kinase, thereby inhibiting the activation of transcription factors such as NF-κB and modulating the cellular inflammatory response (Chen et al., 2014). It also promotes the proliferation of T lymphocytes and inhibits host cell apoptosis. Additionally, in animal models, it upregulates the expression of iNOS and enhances the production of NO, thereby strengthening the immune response of macrophages (Cinatl et al., 2003). Furthermore, glycyrrhizin can suppress viral gene expression and replication, inhibit the production of inflammatory cytokines, reduce viral adsorption and entry, and modulate the host immune response (Pastorino et al., 2018; Sasaki et al., 2002).

### 3.5 Anti-fibrotic effects

Liver fibrosis results from excessive accumulation of extracellular matrix (ECM) following chronic liver injury. HSCs, the primary producers of ECM, play an indispensable role in the pathogenesis of liver fibrosis (Hung et al., 2022). Studies have shown that glycyrrhizin inhibits the proliferation and activation of HSCs and reduces collagen synthesis and deposition. This mechanism may be associated with the inhibition of pro-fibrotic signaling pathways such as Transforming Growth Factor (TGF)-β1/Mothers Against Decapentaplegic Homolog (Smad) (Kisseleva and Brenner, 2021).

The pathogenesis of liver fibrosis is complex, with sustained HSC activation and ECM deposition due to chronic liver injury being major contributors (Horn and Tacke, 2024). Evidence from *in vitro* and animal models has revealed multiple pathways by which glycyrrhizin inhibits HSC activation. Activation of HSCs is marked by upregulation of α-smooth muscle actin (α-SMA) (Figure 4). In CCl_4_-induced mouse models, Glycyrrhizin directly targets and binds to AKR7A2, enhancing its antioxidant function, and synergizes with cytoglobin (CYGB) to effectively clear ROS in HSCs, thereby alleviating oxidative stress. This process ultimately suppresses HSC activation (reducing α-SMA expression), inhibits the mRNA expression of type I and III collagen, reduces the production and deposition of ECM, mitigates fibrotic tissue hyperplasia and pseudolobule formation, and thereby reverses liver fibrosis (Wang Q. et al., 2024; Liang et al., 2015); In a mouse model of hepatic fibrosis established in C57BL/6 male mice, 18β-GA inhibits the activity of peroxiredoxin 1 (PRDX1) and peroxiredoxin 2 (PRDX2) by binding to reactive cysteine residues, inducing ROS-mediated apoptosis in HSCs (Zhang et al., 2022).

Recent studies using both *in vitro* and *in vivo* models indicate that glycyrrhizin directly targets and binds to the RNA-binding protein CUG-binding protein 1(CUGBP1), activating the Interferon (IFN)-γ/Signal Transducer and Activator of Transcription (STAT)1/Smad7 signaling pathway and thereby inhibiting HSC activation, ultimately exerting anti-fibrotic effects (Guo et al., 2023). Specifically, when glycyrrhizin binds to and inhibits CUGBP1, it suppresses its role in promoting IFN-γ mRNA degradation (Wu et al., 2016). This leads to an increase in serum IFN-γ levels. IFN-γ binding to its receptor activates the downstream Janus Kinase (JAK)-STAT pathway, resulting in the phosphorylation of STAT1 (p-STAT1) (Miyazawa and Miyazono, 2017). Activated p-STAT1 enters the nucleus and acts as a transcription factor to upregulate Smad7 expression. Smad7 is a key endogenous inhibitor of the TGF-β/Smad signaling pathway (Liu Z. et al., 2022). Upregulation of Smad7 competitively inhibits Smad2/Smad3 phosphorylation and signal transduction downstream of TGF-β1, thereby blocking its pro-fibrotic effects (Guo et al., 2023).

Moreover, glycyrrhizin can inhibit the expression of fibrosis-related factors such as Connective Tissue Growth Factor (CTGF), Matrix Metalloproteinase (MMP2), and MMP9, reducing basement membrane disruption and inflammatory cell infiltration (Liang et al., 2015).

## 4 Clinical applications and efficacy evaluation

### 4.1 Viral hepatitis

High mobility group box 1 (HMGB1) is a damage-associated molecular pattern (DAMP) molecule that promotes inflammatory responses in viral hepatitis. The hepatoprotective effect of glycyrrhizin is mediated by inhibition of the HMGB1–Matrix Metalloproteinase (TLR4) signaling axis. By blocking HMGB1, glycyrrhizin alleviates immunopathological liver injury and significantly reduces serum alanine aminotransferase (ALT) levels, hepatic necrosis, and inflammatory cell infiltration (Shi X. et al., 2020).Chronic hepatitis B is an infectious disease caused by the hepatitis B virus and represents one of the major global causes of cirrhosis and hepatocellular carcinoma (Mak et al., 2020). Although entecavir, as a first-line antiviral agent, effectively inhibits viral replication, its efficacy in improving liver function remains limited. Therefore, GLPS are often used adjunctively in combination with entecavir in clinical practice to enhance liver function recovery and anti-inflammatory effects. A high-quality network meta-analysis showed that combination therapy with various GLPS and entecavir results in greater improvement in liver function compared to entecavir monotherapy. Among them, MgI injection demonstrated the highest overall response rate and the most significant improvement in aspartate aminotransferase (AST), whereas CG injection was most effective in reducing ALT levels (Gao et al., 2023). Additionally, DG combined with nucleos(t)ide analogues significantly reduced total bilirubin levels, with particularly notable effects during the early phase of treatment (Liu Y. et al., 2022). Clinical studies have shown that glycyrrhizin injection therapy can successfully reduce the incidence of hepatocellular carcinoma and ALT levels in patients with chronic liver disease related to hepatitis C; when combined with conventional Western medicine in the treatment of acute icteric hepatitis, CG injection effectively improved patients’ liver function parameters, markedly reducing total bilirubin, ALT, and AST levels (Ikeda et al., 2006; van Rossum et al., 2001; Manns et al., 2012) (Table 3). It was also more effective than conventional Western medicine alone in overall clinical symptom improvement and shortened the duration of jaundice by approximately 6 days (Liang et al., 2022).

**TABLE 3:** Summary of key clinical trials on glycyrrhizin preparations in liver disease.

Author (Year)	Design	Sample size (N)	Country	Patient	Intervention	Comparator	Primary outcome measures	Main findings	References
Yang (2020)	Randomized controlled trial (RCT)	52	China	Children with acute icteric hepatitis	Compound Glycyrrhizin Injection plus basic treatment	Basic treatment	TB, AST, ALT	Liver function indicators of the observation group were significantly lower	Yang (2020)
Yao et al. (2022)	Retrospective cohort study	304	China	DILI patients	compound glycyrrhizin injection	Supportive or no treatment	ALT and AST levels	CG injections are effective in reducing ALT and AST levels in DILI patients	Yao et al. (2022)
Manns et al. (2012)	RCT	379	German	Chronic hepatitis C patients	glycyrrhizin	placebo	Proportion of patients with ≥50% ALT reduction	GL reduces ALT	Manns et al. (2012)
Ikeda et al. (2025)	a cohort study	1249	Japan	Chronic hepatitis C patients	glycyrrhizin	placebo	Crude carcinogenesis rates	Glycyrrhizin injection therapy significantly decreased the incidence of hepatocellular carcinoma	Ikeda et al. (2025)
van Rossum et al. (2001)	RCT	72	European	Chronic hepatitis C patients	Glycyrrhizin	placebo	Serum ALT	Glycyrrhizin treatment induces a significant ALT decrease	van Rossum et al. (2001)
Wang et al. (2022b)	RCT	80	China	Patients with chronic drug- or herb-induced liver injury	methylprednisolone plus glycyrrhizin	glycyrrhizin	The proportion of patients with sustained biochemical response	Corticosteroid plus glycyrrhizin therapy can achieve both biochemical response and histological improvements	Wang et al. (2022b)
Wang et al. (2019)	RCT	174	China	Patients with acute drug-induced liver injury	magnesium isoglycyrrhizinate	tiopronin	The proportion of ALT normalization	The experimental group’s ALT normal rate was significantly higher than that of the control group	Wang et al. (2019)
Zhang (2023)	RCT	98	China	Alcoholic cirrhosis patient	Reduced glutathione plus CG	Reduced glutathione	Liver function indicators, liver fibrosis indicators	The serum levels of AST, GGT, ALT, TBIL, IV-C, -III in the experimental group were lower	Zhang (2023)
Han (2024)	RCT	160	China	Patients with ALD	CG plus ligustrazine	ligustrazine	The parameters of hepatic function and fibrosis	The parameters of hepatic function, fibrosis, TGFβ1,IL-6,Leptin,TNF-α of combined treatment group were obviously improved	Han (2024)
Abdullah et al. (2023)	RCT	212	Pakistan	Patients with MASLD	Glycyrrhiza Glabra	vitamin E	Fibrosis scores and liver transaminases	The experimental group had lower fibrosis scores and ALT levels.	Abdullah et al. (2023)
Geivandova et al. (2025)	RCT	225	Russia	Patients with MASLD	ursodeoxycholic acid (UDCA) and GA	UDCA	Aminotransferase level	More than a twofold reduction in ALT and AST levels was achieved	Geivandova et al. (2025)
Bi et al. (2023)	Prospective Cohort Study	377	China	Patients with autoimmune hepatitis	GLPS	hormone	the ratio of sustained biochemical responses the total bile acid	The cumulative biochemical remission rate showed no significant difference the total bile acid in the hormone group was significantly higher	Bi et al. (2023)

*Abbreviations:* ALD, alcoholic liver disease; ALT, alanine aminotransferase; AST, aspartate aminotransferase; CG, compound glycyrrhizin; DILI, drug-induced liver injury; GA, glycyrrhetinic acid; GGT, gamma-glutamyl transferase; GL, glycyrrhizin; GLPS, glycyrrhizin preparation; IL-6, interleukin-6; IV-C, type IV, collagen; MASLD, metabolic dysfunction-associated steatotic liver disease; RCT, randomized controlled trial; TB, total bilirubin; TBIL, total bilirubin; TGFβ1, transforming growth factor beta 1; TNF-α, tumor necrosis factor alpha; UDCA, ursodeoxycholic acid; III, type III, collagen.

### 4.2 Drug-induced liver injury (DILI)

Drug-induced liver injury (DILI) refers to liver damage caused by exposure to medications. Numerous drugs and herbs have been reported to cause DILI, including antituberculosis agents, acetaminophen, anticancer drugs, and oral contraceptives (Singh et al., 2015). GLPS are recommended as first-line treatments for DILI in multiple domestic and international guidelines. Their potent anti-inflammatory and membrane-stabilizing effects effectively block the cascade of hepatocyte necrosis triggered by drugs and their metabolites, rapidly reducing ALT and AST levels (Yao et al., 2022). For example, clinical studies have shown that the combination of glucocorticoids and GL for the treatment of chronic DILI can simultaneously achieve biochemical response and histological improvement, with good safety (Wang JB. et al., 2022). MgIG may be superior to tiopronin in the treatment of acute drug-induced liver injury (Wang et al., 2019). A recent network meta-analysis on different hepatoprotective drugs for drug-induced liver injury found that CG was most effective in reducing AST levels, MgIG performed best in lowering ALT, and GLPS were not as effective as polyene phosphatidylcholine in reducing total bilirubin (TBIL) (Li et al., 2023) (Table 3). In recent years, research on the application of glycyrrhizin in DILI has remained a focus of active research.

#### 4.2.1 Chemical drug-induced liver injury

Glycyrrhizin activates the β-catenin signaling pathway, upregulates proliferation-related proteins (such as Proliferating Cell Nuclear Antigen (PCNA), Cyclin D1, and Cyclin-Dependent Kinase (CDK) 4), promotes hepatocyte proliferation and liver tissue regeneration, thereby effectively alleviating acetaminophen-induced acute liver injury and significantly reducing acetaminophen (APAP)-elevated serum ALT and AST levels (Cao et al., 2025). DG combined with cysteine hydrochloride (CH) exerts hepatoprotective effects against acetaminophen-induced DILI, primarily by inhibiting oxidative stress and activating the Kelch-like ECH-associated protein (Keap)1/Nuclear factor erythroid 2-related factor (Nrf)2/Antioxidant Response Element (ARE) pathway (Li et al., 2022). MgIG mitigates APAP-induced liver injury by promoting mitochondrial biogenesis (Xia et al., 2025). However, there are few related clinical studies on this aspect.

MgIG counteracts antituberculosis drug-induced liver injury through multiple mechanisms. Antituberculosis drugs (HRZE) cause gut microbiota dysbiosis, particularly reducing the abundance of probiotics such as *Lactobacillus*, which is negatively correlated with liver injury markers (e.g., AST, ALT) (Li G. et al., 2024). MgIG significantly restores the abundance of *Lactobacillus*; it upregulates the expression of tight junction proteins (e.g., ZO-1 and occludin) in colon tissue, repairs the intestinal epithelial barrier, reduces serum FD4 (fluorescein isothiocyanate–dextran) levels, decreases intestinal permeability, prevents bacterial and metabolite translocation, and lowers serum LPS levels; MgIG also downregulates the mRNA expression of TLR2, TLR4, and NF-κB in the liver, thereby reducing the production of pro-inflammatory cytokines (e.g., TNF-α, IL-6) and alleviating liver tissue inflammation and oxidative stress (Chopyk and Grakoui, 2020; Cao et al., 2019; Gong et al., 2022a). There is already extensive clinical research on the application of GLPS in liver injury caused by anti-tuberculosis drugs. A recent network meta-analysis, which summarized 97 randomized controlled trials, concluded that MgIG injection may be the optimal choice, while ammonium glycyrrhizinate capsules are the least favorable option among all clps (Gong et al., 2022b). This also explains why many basic research studies focus on MgIG rather than other GLPS.

DG primarily alleviates amphotericin B-induced liver injury through dual mechanisms involving inhibition of oxidative stress and apoptosis (He et al., 2024). Specifically, it effectively clears excess reactive oxygen species in the liver and enhances the liver’s antioxidant defense capacity by increasing the activity of key antioxidant enzymes such as superoxide dismutase, catalase, and reduced glutathione, while reducing the level of malondialdehyde (a lipid peroxidation product), thereby mitigating oxidative stress damage (He et al., 2024; Zhao et al., 2022). In terms of anti-apoptosis, DG regulates the Bcl-2/Bax protein balance, inhibits the mitochondrial apoptosis pathway, thereby suppressing the cascade activation of Caspase-8, Caspase-9, and Caspase-3 as well as Poly (ADP-ribose) polymerase (PARP) cleavage; additionally, it inhibits the nuclear translocation of caspase-independent apoptosis-inducing factor (AIF), thus blocking programmed hepatocyte death through multiple pathways (He et al., 2024; Zhao et al., 2022; Luo et al., 2021). Although there have been some recent mechanism-related studies, relevant clinical research is still lacking at present.

#### 4.2.2 Hepatic injury caused by herbal medicines and their preparations

GA effectively alleviates triptolide (TGT)-induced acute liver injury through multi-target and multi-pathway synergistic mechanisms. Firstly, GA significantly upregulates key drug-metabolizing enzyme genes (e.g., *Cyp2b13*, *Cyp2c69*, *Cyp3a44*, *Cyp3a16*, and *Fmo3*) that are downregulated by TGT, thereby enhancing liver detoxification capacity (Shi et al., 2021). Secondly, GA reverses TGT-induced lipid metabolism disorders, particularly restoring phosphatidylcholine (PC) and phosphatidylethanolamine (PE) metabolism, thereby maintaining hepatocyte membrane integrity and stabilizing related signal transduction functions, which aligns with the lipid remodeling phenomenon observed in TGT-induced liver injury (Shi et al., 2021; Xie et al., 2016). Additionally, GA exhibits potent antioxidant capacity, significantly increasing the activity of superoxide dismutase (SOD) and glutathione peroxidase (GSH-Px) in serum, thereby mitigating oxidative damage (Shi et al., 2021). GA also alleviates oxidative stress via Pyruvate Kinase M2-mediated mechanisms and inflammation, reducing hepatocyte apoptosis and thus mitigating TGT (anti-rheumatic)-induced acute liver injury (Wang Q. et al., 2022). Ultimately, these molecular-level regulations collectively result in significant improvement in liver function: GA effectively reduces TGT-induced elevations in serum ALT, AST, Alkaline Phosphatase (ALP), and TBIL levels and alleviates pathological liver tissue damage. There are many clinical studies in China investigating the combined application of GA and tripterygium. A systematic review concluded that when CG is used in combination with tripterygium glycosides, it can significantly reduce the incidence of abnormal liver function (Bing, 2021).

Diosbulbin B, a compound isolated from the medicinal plant *Dioscorea*, can cause severe liver injury. Glycyrrhizin inhibits the metabolism of diosbulbin B (Yang et al., 2024), promotes bile acid efflux by increasing the expression of Nrf2/Farnesoid X Receptor- Bile Salt Export Pump (FXR-BSEP)/Multidrug Resistance-Associated Protein (MRP)2/P-glycoprotein/UDP-glucuronosyltransferase 1A1(UGT1A1), modulates gut microbiota, alleviates inflammation and oxidative stress, and improves diosbulbin B-induced liver injury (Wang et al., 2025).

#### 4.2.3 Hepatic injury induced by other substances

GA primarily alleviates iron overload-induced liver injury in mice by modulating the gut-liver axis: DG partially reduces iron deposition and ferrous ion levels in the liver of iron-overloaded mice, thereby mitigating oxidative damage; DG also ameliorates gut microbiota dysbiosis, repairs intestinal barrier damage, and inhibits TLR4/NF-κB/NLRP3 pathway-mediated liver inflammation induced by iron overload (Liu Y. et al., 2024; Liu et al., 2013; Xu et al., 2021). Additionally, DG modulates bile acid metabolism disorders in iron-overloaded mice and reduces bile acid accumulation in the liver (Liu Y. et al., 2024). MgIG partially alleviates cadmium-induced c-Jun N-terminal kinase (JNK)-mediated hepatocyte apoptosis by reversing Protein Phosphatase (PP) 2A inactivation (Chen et al., 2021).

Another study showed that monoammonium glycyrrhizinate (MAG) exerted hepatoprotective effects against aluminum and fructose (FRUAL)-induced liver injury that surpassed those of the gold standard silymarin, suggesting potential future replacement of silymarin (Zakaria et al., 2020). However, the mechanisms underlying these effects remain unclear and require further investigation.

### 4.3 Alcohol-associated liver disease (ALD) and MASLD

Through its anti-inflammatory, antioxidant, and membrane-stabilizing properties, glycyrrhizin mitigates liver damage caused by alcohol and its metabolite acetaldehyde, improving biochemical markers in patients with alcoholic hepatitis (Wang X. et al., 2024; Huo et al., 2019) (Table 3). DG alleviates alcohol-induced liver injury by modulating the DDX5/STAT1 signaling axis. Specifically, it reverses the alcohol-induced decrease in DDX5 protein expression. The upregulated DDX5 then recruits Protein Inhibitor of Activated STAT (PIAS)1, an inhibitory protein of STAT1, thereby blocking STAT1 phosphorylation and activation. This ultimately inhibits hepatic lipid deposition, reduces oxidative stress, and alleviates inflammatory responses, thereby exerting hepatoprotective effects (Wang X. et al., 2024; Zha et al., 2023). In the pathogenesis of alcoholic fatty liver disease, macrophages play a key role. Chronic alcohol intake activates intrahepatic macrophages, releasing inflammatory cytokines such as TNF-α and IL-1β, which promote liver inflammation, lipid accumulation, and fibrosis (Zhou et al., 2022; Zhang et al., 2019). Glycyrrhizin, particularly its derivative DG, alleviates the pathological progression of alcoholic fatty liver by regulating the Src Homology Region 2 Domain-Containing Phosphatase (SHP)1/Spleen Tyrosine Kinase (SYK) signaling pathway in macrophages, thereby inhibiting hepatic oxidative stress, inflammatory responses, and pyroptosis (Liu Q. et al., 2024). Multiple Chinese clinical studies have shown that the combination of CG and reduced glutathione is significantly more effective than reduced glutathione alone in the treatment of alcoholic cirrhosis (Zhang, 2023) (Table 3). In addition, the combination of puerarin, ligustrazine, and glycyrrhizic acid preparations can significantly improve patients' liver function and fibrosis indicators (Han; Hui et al., 2014).

Metabolic Dysfunction-Associated Steatotic Liver Disease is a common chronic liver condition in which lipid homeostasis, insulin resistance, and glucose homeostasis play critical roles (Chao et al., 2019). GA primarily exerts its multi-faceted protective effects against MASLD by activating the FXR signaling pathway. It downregulates Sterol Regulatory Element-Binding Protein (SREBP)-1c, Fatty Acid Synthase (FAS), Stearoyl-CoA Desaturase (SCD)1, Phosphoenolpyruvate Carboxykinase (PEPCK), Glucose-6-Phosphatase (G6Pase), and Apolipoprotein (Apo) C-III, while upregulating PPARα, Carnitine Palmitoyltransferase (CPT) 1α, Acyl-CoA Dehydrogenase, Short Chain (ACADS), Pyruvate Dehydrogenase (PDase), Glycogen Synthase Kinase (GSK) 3β, and Apo C-II (Ma et al., 2013; Pineda Torra et al., 2003; Sun et al., 2017). This enhances fatty acid β-oxidation, promotes triglyceride breakdown, and regulates lipid and glucose metabolism (Sun et al., 2017; Wang et al., 2016). As a novel Aldo-Keto Reductase Family 1 Member (AKR1)B10 inhibitor, GA promotes retinoic acid synthesis and restores retinol metabolism balance in MASLD mouse models (Shi L. et al., 2020). Glycyrrhizin also exerts therapeutic effects on MASLD by modulating gut microbiota (Table 3). It significantly ameliorates high-fat diet-induced structural dysbiosis of the gut microbiota by reducing the abundance of harmful bacteria positively correlated with hepatic lipid deposition (e.g., Lachnospiraceae and Collinsella) and increasing the abundance of beneficial bacteria (e.g., Ruminococcus and Turicibacter), thereby reducing hepatic lipogenesis and ultimately alleviates liver steatosis and lipid accumulation (Wang S. et al., 2022; Salman et al., 2024). Clinical studies have shown that licorice is more effective than vitamin E in improving liver fibrosis and reducing ALT levels (Abdullah et al., 2023). GA combined with ursodeoxycholic acid can significantly reduce liver enzyme levels within 12 weeks and enhance the effectiveness of treatment for MASLD. (Geivandova et al., 2025).

### 4.4 Autoimmune hepatitis (AIH)

Autoimmune hepatitis (AIH) is a type of liver inflammation characterized by the immune system attacking hepatocytes, leading to chronic liver injury (Terziroli Beretta-Piccoli et al., 2022; Li M. et al., 2024). The current first-line treatment for AIH involves a combination of glucocorticoids and azathioprine (Reau et al., 2024). For patients who require glucocorticoid dose reduction or cannot tolerate standard therapy due to side effects, GLPS, particularly CG, can serve as an adjunctive or alternative treatment option to help control the disease and reduce glucocorticoid-related adverse effects (Bi et al., 2023).

The concanavalin A (ConA)-induced liver injury model is commonly used to study AIH. Studies have shown that glycyrrhizin alleviates ConA-induced acute liver injury by modulating the function of monocyte-derived macrophages (Lu et al., 2024). On one hand, it improves liver injury markers, reduces serum ALT and AST levels, and attenuates hepatic necrosis and inflammatory cell infiltration. On the other hand, glycyrrhizin ameliorates the inflammatory state by regulating the immune microenvironment. This is achieved through increasing anti-inflammatory Monocyte-Derived Macrophage subset (MoMF)-1 cells, decreasing pro-inflammatory MoMF-3 cells, and influencing MoMF cytokine secretion, which collectively result in reduced levels of pro-inflammatory factors such as IL-6, C-C Motif Chemokine Ligand (CCL)2, and C-X-C Motif Chemokine Ligand (CXCL)9 (Lu et al., 2024; Liu M. et al., 2022; Wang K. et al., 2022).

In patients with mild AIH, GLPS demonstrate long-term efficacy comparable to standard glucocorticoid therapy but with a superior safety profile and a significantly lower incidence of adverse effects (Bi et al., 2023) (Table 3). There is no significant difference in biochemical remission rates or the degree of improvement in liver function indicators between the two treatments. However, the safety profile of the glycyrrhizin group is notably better. Typical glucocorticoid-related side effects, such as osteoporosis, diabetes, hypertension, and peptic ulcers, which are common in the glucocorticoid group, are rare or absent in patients receiving glycyrrhizin. (Bi et al., 2023).

### 4.5 Acute liver injury induced by lipopolysaccharide

LPS, a component of the cell wall of Gram-negative bacteria also known as endotoxin, plays a significant role in the development of hepatitis and acute liver injury (ALI) by triggering the overexpression of pro-inflammatory cytokines. When LPS enters the bloodstream, it strongly activates the immune system, leading to the massive release of pro-inflammatory cytokines and the generation of large amounts of ROS, ultimately resulting in hepatocyte death and acute liver injury (Zhang G. et al., 2025).

A novel nano-delivery system named GA/PPC-LNP, with glycyrrhizin as a key component, has been developed. On one hand, this system utilizes the inherent anti-inflammatory and antioxidant activities of glycyrrhizin and polyene phosphatidylcholine. On the other hand, it efficiently delivers p65 siRNA to target and silence NF-κB, thus reducing hepatocyte injury markers (ALT/AST), suppressing the release of inflammatory factors, and significantly ameliorating acute liver injury by targeting this central inflammatory pathway (Yin et al., 2023). DG exerts a protective effect in LPS-induced acute liver injury by activating the Nrf2/ARE pathway, enhancing the liver’s antioxidant capacity, and indirectly inhibiting inflammatory responses. Its combination with cysteine further enhances antioxidant efficacy, demonstrating a synergistic effect—particularly in promoting glutathione (GSH) synthesis (Chu et al., 2020). HMGB1, an important inflammatory mediator, is significantly upregulated under LPS stimulation. In LPS-activated macrophages, the expression of Phosphorylated Phosphatidylinositol 3-Kinase (p-PI3K) and Phosphorylated Mammalian Target of Rapamycin (p-mTOR) increases (Chen et al., 2004; Xue et al., 2017; Bonifazi et al., 2010; Choi et al., 2013). Glycyrrhizin markedly alleviates inflammation, oxidative stress, and apoptosis in LPS-induced acute liver injury by inhibiting HMGB1 and its downstream PI3K/mTOR signaling pathway, thereby improving liver tissue structure and function (Shen et al., 2020).

### 4.6 Other liver diseases

In the early or intermediate stages of liver failure, GLPS can be used as part of comprehensive treatment to control inflammation and delay disease progression. Glycyrrhizin-mediated liver-targeted sodium alginate nanogels may represent a promising approach for the treatment of acute liver failure (Zhao et al., 2021). Portal hypertension (PHT) is a complication of liver disease, involving macrophage activation and superoxide dismutase 3 (SOD3) in its pathogenesis. DG reduces portal pressure by restoring SOD3 activity in the portal area, counteracting oxidative stress in portal vein macrophages, and maintaining the bioavailability of nitric oxide and prostacyclin (PGI_2_) (Zhao et al., 2022).

During lung cancer treatment, gefitinib activates the p53/p21 pathway in hepatocytes, leading to cell cycle arrest and subsequent liver injury. Glycyrrhizin alleviates liver injury by precisely inhibiting the overactivation of this pathway in normal hepatocytes, which reverses cell cycle blockade and restores normal hepatocyte proliferation and function through downregulating p53/p21 and upregulating Cyclin D1, all without compromising the antitumor efficacy of gefitinib via the same pathway in cancer cells (Li et al., 2025). However, it is regrettable that clinical research in this area remains blank at present.

### 4.7 Glycyrrhizin and other hepatoprotective agents

A variety of hepatoprotective agents are currently used in clinical practice, including silymarin, N-acetylcysteine (NAC), ursodeoxycholic acid (UDCA), as well as polyene phosphatidylcholine (PPC), bicyclol, S-adenosylmethionine (SAMe), and glucocorticoids. While these agents share overlapping indications with glycyrrhizin, their mechanisms of action are distinct yet potentially complementary, offering opportunities for synergistic combination strategies in the management of liver diseases (Li et al., 2021). For example, in activated hepatic stellate cells, 18α-glycyrrhizin demonstrated superior anti-fibrotic efficacy compared to silymarin under therapeutic condition (Hosseini et al., 2016). Chen Y et al. evaluated the pharmacoeconomics of three therapeutic schemes in treating Anti-TB DILI of 225 newly treated TB patients. The results revealed that the efficacy of bicyclol in the treatment of Anti-TB DILI was superior to silybin and DG, with good safety (Chen et al., 2018). A randomized double blind clinical trial concluded that silymarin is a safe herbal medication but have no effect on hepatic toxicity of anti-tuberculosis drugs (Marjani et al., 2019).

Combination regimens involving different hepatoprotective agents have emerged as a key research focus in recent years. Preclinical studies have demonstrated that both glycyrrhizin and silymarin exert hepatoprotective effects against oxidative stress, and their combination may produce synergistic benefits by reducing serum transaminases and enhancing antioxidant enzyme activities (Chen et al., 2014). In the setting of acetaminophen-induced acute liver injury, NAC remains the standard of care; however, emerging evidence from a murine model suggests that co-administration of glycyrrhizin with NAC significantly reduces hepatocyte necrosis and improves survival compared to NAC alone, potentially by combining NAC’s detoxification mechanism with glycyrrhizin’s inhibition of the pro-inflammatory mediator HMGB1 (Minsart et al., 2020). Furthermore, a randomized controlled trial in patients with chronic hepatitis C demonstrated that combination therapy with UDCA and glycyrrhizin produced significantly greater reductions in serum aminotransferases and γ-glutamyl transpeptidase (γ-GTP) compared to glycyrrhizin alone, without affecting HCV RNA levels, indicating a hepatoprotective rather than antiviral synergy (Tsubota et al., 1999). These comparative insights help contextualize glycyrrhizin within the existing therapeutic landscape and highlight opportunities for rational combination strategies.

## 5 Adverse reactions

**FIGURE 5:**
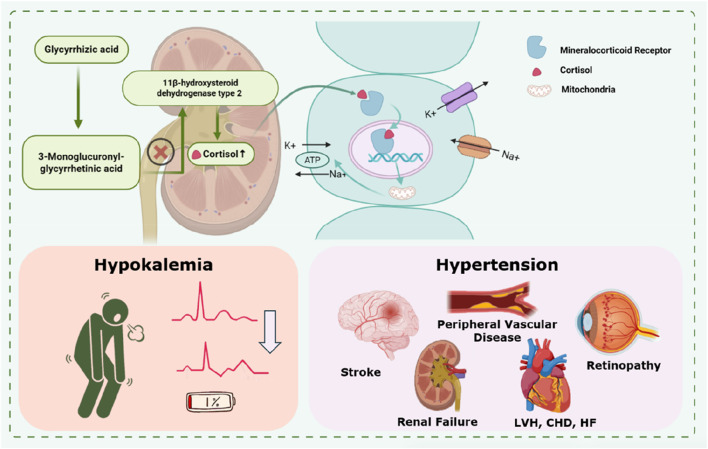
Mechanism of GA-induced pseudo hyperaldosteronism:Inhibition of 11β-HSD2.

Pseudohyperaldosteronism is the most common adverse reaction. A literature review examining the relationship between glycyrrhiza dosage and pseudoaldosteronism found that the mean incidence was 1.0% at a daily dose of 1 g, increasing to 1.7% at 2 g/day, 3.3% at 4 g/day, and reaching 11.1% at 6 g/day (MANTANI et al., 2015). In patients receiving long-term administration of glycyrrhizin or GA, serum levels of 3MGA are significantly elevated (Kato et al., 1995). Type II 11β-hydroxysteroid dehydrogenase (11β-OHSD2) catalyzes the conversion of glucocorticoids to an inactive state and prevents glucocorticoids from accessing the mineralocorticoid receptor, thereby maintaining the specificity of the mineralocorticoid receptor for aldosterone (Zhang, 2011). 3MGA is a potent inhibitor of 11β-OHSD2. Elevated serum levels of 3MGA inhibit 11β-OHSD2, leading to increased cortisol levels. Cortisol then binds to the mineralocorticoid receptor, producing symptoms similar to aldosterone excess, including water and sodium retention, hypokalemia, hypertension, and edema (Ohtake et al., 2007) (Figure 5). When combined with antihypertensive or diuretic agents, the risk of hypokalemia is increased (Ravi and Calum, 2020). Severe hypokalemia can destabilize the membrane potential of skeletal muscle cells, resulting in muscle weakness and paralysis; in severe cases, cellular damage may lead to rhabdomyolysis. Hypokalemia also directly affects the electrophysiological activity of myocardial cells, causing electrocardiographic abnormalities (such as U waves and prolonged QT interval) and significantly increasing the risk of fatal arrhythmias (e.g., torsades de pointes) (Elinav and Chajek-Shaul, 2003; van den Bosch et al., 2005; Eriksson et al., 1999; Panduranga and Al-Rawahi, 2013).

In addition to blood pressure elevation mediated through renal mechanisms, glycyrrhizin also acts directly on the cardiovascular system in multiple ways. On one hand, metabolites of glycyrrhizin enhance vascular contraction in response to pressor hormones such as angiotensin II, thereby increasing vascular tension. On the other hand, glycyrrhizin inhibits endothelial NO synthesis, reducing vasodilation. Together, these effects contribute to hypertension (Ceccuzzi et al., 2023; Deutch et al., 2019). Furthermore, animal studies have shown that injecting GA into the brains of rats elevates blood pressure, suggesting a potential central pressor mechanism independent of 11β-HSD2 inhibition (Ceccuzzi et al., 2023).

GA also exerts several effects on the endocrine and reproductive systems. It exhibits estrogen-like activity by binding to estrogen receptors, influencing calcium balance and uterine responsiveness. It also suppresses androgen activity and reduces testosterone levels. Additionally, GA may inhibit 5β-reductase and 3β-hydroxysteroid dehydrogenase in the liver, slowing the degradation of aldosterone and affecting its metabolism (Latif et al., 1990; Mattarello et al., 2006).

The long-term safety of GLPS warrants attention due to their widespread clinical use as hepatoprotective and anti-inflammatory agents. A literature analysis of severe hypokalemia induced by GLPS reported that CG tablets were the most frequently implicated agent, with a median serum potassium level of 1.8 mmol/L. Severe hypokalemia occurred at a median of 60 days after treatment initiation (range 4 days to over 1 year), and recovery was achieved at a median of 7 days after drug discontinuation and potassium supplementation (Liu Sitong et al., 2021). A case report described a 50-year-old female patient who developed pseudoaldosteronism complicated by hypokalemic rhabdomyolysis after irregular oral administration of GLPS for approximately 2 years. Her serum potassium dropped to 1.9 mmol/L, and creatine kinase levels exceeded 2,200 U/L; symptoms gradually resolved after drug withdrawal and potassium repletion (Xue, 2021).

## 6 Limitations

Despite the comprehensive delineation of glycyrrhizin’s multifaceted mechanisms in preclinical models, it is imperative to acknowledge that most of these pathways have been primarily characterized *in vitro* and animal studies. While the clinical studies reviewed here consistently support the hepatoprotective efficacy of GA, these findings must be interpreted with consideration of substantial heterogeneity and methodological limitations. Study designs vary widely, ranging from RCTs to retrospective analyses; most RCTs inadequately report randomization, allocation concealment, or blinding, introducing risks of selection and detection bias that may inflate treatment effects. The majority of studies are small and single-center, leading to unstable effect estimates, and increased risk of false-positive findings. Geographically, the evidence is heavily concentrated in China and other East Asian countries, with minimal data from Western populations, which limits generalizability across ethnicities, healthcare systems, and regulatory contexts. Additional heterogeneity arises from diverse interventions (different formulations, routes, dosages, durations) and inconsistent comparators, compounded by non-uniform outcome definitions and diagnostic criteria for liver injury.

Equally important, all included studies reported positive findings, with no clearly negative or null results identified. While this may partly reflect the genuine pharmacological activity of GL, the absence of negative studies raises the strong possibility of publication bias, particularly given the geographic concentration of literature. Furthermore, despite uniformly positive conclusions, considerable heterogeneity exists in effect sizes; smaller studies tend to report larger treatment effects, suggesting small-study effects. In summary, while the existing evidence points toward a beneficial role for GA in liver disease, the consistent positivity should be interpreted with caution, likely reflecting a combination of genuine therapeutic activity, methodological limitations, and publication bias. Future research should prioritize large, multicenter, rigorously designed RCTs with standardized outcomes, and efforts to publish both positive and negative findings are essential to establish a balanced evidence base.

## 7 Summary

GLPS, derived from the traditional Chinese medicine licorice, represent a modern therapeutic option for liver diseases. Owing to their multi-target and synergistic pharmacological mechanisms, such as anti-inflammatory, membrane-stabilizing, antioxidant, anti-apoptotic, and immunomodulatory effects, they have demonstrated clear efficacy in the clinical treatment of various liver conditions, including viral hepatitis, drug-induced liver injury, and alcoholic liver disease. These agents have become foundational agents in adjunctive liver therapy. However, the regulatory status and clinical approval of GLPS vary considerably across regions. In Japan, the intravenous formulation Stronger Neo-Minophagen C (SNMC) has been approved for chronic hepatitis for over 4 decades, with long-term studies demonstrating its efficacy in reducing ALT levels and preventing hepatocellular carcinoma (Kumada, 2002; Arase et al., 1997). In China, various GLPS are widely used and have been officially approved for treating acute drug-induced liver injury (Li et al., 2019). In contrast, regulatory approval in Western countries remains limited: in the United States, glycyrrhizin is primarily recognized as a food sweetener rather than a therapeutic agent, while in Europe, its use is largely confined to traditional medicine and flavoring. This heterogeneous regulatory landscape underscores the need for additional high-quality clinical trials to support broader international approval.

Looking ahead, future research on GLPS should advance along several interconnected fronts. At the formulation level, developing novel drug delivery systems through structural optimization and nanotechnology represents a key priority, with glycyrrhizin-based nanocarriers already demonstrating improved oral bioavailability and hepatic targeting in preclinical studies (Li et al., 2026; Xu et al., 2025). Building on these advances, combination strategies with antiviral and antifibrotic agents warrant systematic clinical investigation—recent studies have shown that glycyrrhizin combined with entecavir significantly improves liver function and reduces fibrosis markers in chronic hepatitis B, while its combination with Wuzhi capsule demonstrates superior efficacy in non-alcoholic steatohepatitis (Li, 2025; Gao, 2025). A recent meta-analysis of 15 RCTs further confirmed the overall benefit of glycyrrhizin-containing regimens on liver enzymes across various liver diseases (Xu et al., 2025; Giangrandi et al., 2024). To inform and optimize such combination approaches, deeper mechanistic understanding is needed; omics and systems biology technologies offer powerful tools to uncover new pathways of action, particularly regarding gut microbiota modulation and the hepatic immune microenvironment (Zhang Y. et al., 2025; Patel et al., 2025). As an “evergreen” in the field of liver disease treatment, GLPS are poised to continue playing a key role and bring further clinical benefits to patients worldwide.
